# Preparation of* Galla Chinensis* Oral Solution as well as Its Stability, Safety, and Antidiarrheal Activity Evaluation

**DOI:** 10.1155/2017/1851459

**Published:** 2017-07-25

**Authors:** Yi Yang, Huihui Luo, Xu Song, Li Yu, Juan Xie, Jiajie Yang, Renyong Jia, Juchun Lin, Yuanfeng Zou, Lixia Li, Lizi Yin, Changliang He, Xiaoxia Liang, Guizhou Yue, Zhongqiong Yin

**Affiliations:** ^1^Natural Medicine Research Center, College of Veterinary Medicine, Sichuan Agricultural University, Chengdu 611130, China; ^2^College of Veterinary Medicine, Sichuan Agricultural University, Chengdu 611130, China; ^3^College of Science, Sichuan Agricultural University, Ya'an 625014, China

## Abstract

**Background of the Study:**

As a widely used traditional medicine,* Galla Chinensis* is rich in tannins. However, there are few detailed studies about pharmaceutical preparations of* Galla Chinensis* tannin extract (GTE). In the present experiments, for better application and to investigate the possibility that* Galla Chinensis* tannin extract can be used as an antidiarrheal drug, we prepared* Galla Chinensis* oral solution (GOS).

**Materials and Methods:**

GOS was prepared with GTE, and its physicochemical and microbiological stability was evaluated. The oral acute toxicity of GOS was calculated by the 50% lethal dose (LD_50_). The antidiarrheal activity was determined in a castor oil-induced diarrhea model in mice through diarrhea symptoms, fluid accumulation ratio, and percentage of distance moved by charcoal meal.

**Results:**

The tannin content of GTE was 47.75%. GOS could endure a high temperature without a significant decrease of tannin content. After storage for six months, the tannin content of GOS was still more than 90%. GOS was determined to be nontoxic. Meanwhile, GOS showed significant antidiarrheal activity in a castor oil-induced diarrhea model in mice (*P* < 0.01).

**Conclusion:**

The results suggested that GOS is an effective and stable antidiarrheal drug that can be used to complement other therapies.

## 1. Introduction

Diarrhea is such a disease that always threatens the health of human beings and many species of animals, especially children under five, and lots of young animals like piglets and newborn (suckling) calves [1–3]. It comes with many typical symptoms, including gastrointestinal motility disorders, soft or liquid stools, increasing frequency of defecation, and abdominal pain [4]. Diarrhea has many types. Secretory diarrhea is a branch of diarrhea, because its typical symptom is excessive fluid secretion. When the balance between absorption and secretion in the small intestine is out of control (usually excessive secretion), secretory diarrhea comes soon [5]. In general, excessive secretion can be caused by inflammation, indigestible food, and toxins that come from medications, plants, animals, and bacteria [6].

Reports had shown that many medicinal plants mitigate diarrhea, such as leaves from* Caesalpinia bonducella* and* Calpurnia aurea,* flaxseed,* Alpinia oxyphylla *Miq.*, Qualea parviflora *Mart., and* Terminalia chebula *[7–12], and most of them have revealed the presence of tannins. It is accepted that tannins have antimutagenic, anticarcinogenic, and antioxidant activities and can inhibit gastric H^+^-K^+^-ATPase activity and CFTR chloride channel which enable it to treat diarrhea [13]. In fact, many medical plants which are rich in tannins were proved effective in diarrhea [14]. Tannin-rich carob pod (which contains 40% tannins) produced an effect in acute-onset diarrhea [15]. Rhubarb tannins extract (which contains 55.69% equivalent of gallic acid) could inhibit MgSO_4_-induced diarrhea [16].* Galla Rhois* tannin extract (tannin accounts for 45.8%) reduced the incidence of diarrhea in postweaning piglets [17].

*Galla Chinensis* contains a generous amount of tannins, which even can reach 50–70% of its weight [18]. It is extremely abundant in China, especially in Sichuan. It is the term used to describe the gall caused by the Chinese aphid (family Pemphigidae) on the* Rhus* leaves of the family Anacardiaceae (mainly* Rhus chinensis* Mill.,* Rhus potaninii* Maxim., and* Rhus punjabensis* var.* sinica* (Diels) Rehd. et Wils.). [19, 20]. Tannin from* Galla Chinensis* is a type of hydrolyzable tannin that consists of a central glucose core, which is surrounded by several gallic acid units, and further gallic acid units can be attached through depside bonding of additional galloyl residues. Structures containing 1 to 14 galloyl residues result from such processes, yielding tri-, tetra-, penta-, hepta-, and nonagalloyl glucose, and others [21]. It is pharmacologically demonstrated that* Galla Chinensis* has astringent, anti-inflammatory, local anesthetic, antipyretic, antiparkinsonian, antiaging, antitumor, antiparasitic, antioxidant, and antibacterial activities [20, 22–24].* Galla Chinensis* has been used for a long time for the treatment of diarrhea, prolonged coughing, and spontaneous perspiration in traditional Chinese medicine [25].

It is found that the acetone extract of* Galla Chinensis *was effective on ETEC enterotoxin-induced diarrhea in mice a decade ago [26]. Other studies showed that a hydrolyzable tannin isolated from* Galla Chinensis* can reduce enterotoxin-induced intestinal fluid secretion in mice [13], indicating a part of the antidiarrheal mechanism of* Galla Chinensis* hydrolyzable tannins.

However, hydrolyzable tannins are not stable because they can easily be hydrolyzed and oxidized [27]. Preparation is a way to solve this problem, and there are few detailed studies on the pharmaceutical preparations of* Galla Chinensis* tannin extract in medicines. For better application and for protecting* Galla Chinensis *tannin-rich extracts from being oxidized, in the current experiments, we prepared an oral solution of tannins extract from* Galla Chinensis *(GOS) and evaluated its stability at two conservation conditions (40 ± 2°C and 60 ± 2°C) for six months and ten days in unopened brown bottles. Moreover, the acute toxicity and antidiarrheal activity of GOS were assessed in mice.

## 2. Materials and Methods

### 2.1. Preparation of* Galla Chinensis* Tannin Extract

*Galla Chinensis* (batch number: D02115E01) was produced by Sichuan Zhongyong Pharmaceutical Co., Ltd. (Chengdu, China). The plant materials were identified by Dr. Lixia Li (Sichuan Agricultural University). A sample of the plant material was deposited at the herbarium of the Natural Medicine Research Center, College of Veterinary Medicine, Sichuan Agricultural University, with the voucher number 2015–0768. The* Galla Chinensis* tannin extract (GTE) was prepared as follows. Briefly, the dried and powdered plant was extracted with boiling distilled water in a ratio of 1 : 15 (w : v) for 2 h, thrice. After filtration, all crude extract was collected. These solutions were purified by chloroform in a ratio of 1 : 1 (v : v) twice. Then, the water phase was collected and mixed with ethylacetate in a ratio of 1 : 2 (v : v). The mixture was shaken in a water bath at 45°C for 30 min and then ethylacetate phase was collected. Distilled water was added and organic solvents were removed from the ethylacetate phase under vacuum. Finally, the tannin extract powder was obtained by lyophilization.

### 2.2. Determination of Tannin Content in GTE

Gallic acid (GA; Solarbio, China) solutions (0.05 mg/mL) in 0 mL, 0.5 mL, 1.0 mL, 2.0 mL, 3.0 mL, 4.0 mL, and 5.0 mL were mixed with 1 mL Folin–Ciocalteu reagent (FCR; Solarbio, China), respectively. The solutions were mixed with 12 mL, 11.5 mL, 11 mL, 10 mL, 9 mL, 8 mL, and 7 mL water severally, and then 29% sodium carbonate (Na_2_CO_3_) was added to each mixture until the liquid volume reached 25 mL. The concentrations of GA here were 0, 0.02, 0.04, 0.06, 0.08, and 0.1 mg/mL, respectively. Finally, after incubation for 30 min at room temperature, each sample was measured at 760 nm using a UV spectrophotometer (UV-2800A, Unic, China) and a standard curve of gallic acid was drawn [25].

The content of tannins in GTE was determined by using the Folin–Ciocalteu method with gallic acid equivalents, according to the methods mentioned in the Chinese Pharmacopoeia [25] by using a UV spectrophotometer. Tannin content was evaluated with the following formula:

(1) Tannin  content=total  phenolic  content−nontannin  polyphenols  content

The total phenolic and nontannin polyphenol contents in GTE were determined as described above. Briefly, a pretreated GTE sample solution in 2 mL was mixed with 1 mL of FCR and 10 mL of water. Then, 29% sodium carbonate (Na_2_CO_3_) was added and left for 30 min at room temperature. The OD_760_ of total phenolics was measured. 0.6 grams of casein was added to the same pretreated GTE sample (25 mL), and then tannins were precipitated by a shaking water bath at 30°C for 1 h before filtration. 1 mL of FCR and 10 mL of water were added to 2 mL of filtrate and 12 mL of 29% sodium carbonate (Na_2_CO_3_). The reaction solution was incubated at room temperature for 30 min and then the absorption value was measured at 760 nm to get the OD of nontannin polyphenols. The results were obtained through comparison with a gallic acid calibration curve.

### 2.3. Preparation of* Galla Chinensis* Oral Solution

*Galla Chinensis* oral solution (GOS) was prepared with different concentrations of GTE (5%, 10%, and 15%), purified water, and appropriate excipients (4% sucrose, 0.25% benzoic acid, 0.05% ethyl* p*-hydroxybenzoate, and 0.2% natrium pyrosulfurosum). The GOS containing different concentrations of GTE (5%, 10%, and 15%) were used in the current study.

### 2.4. Animals

ICR mice (weighing 18–22 g) were purchased from a specific pathogen-free (SPF) facility at Chengdu Dossy Experimental Animals Co., Ltd. (license number SCXK (Sichuan) 2015-030). All of them got enough food and water and were maintained in an animal house at controlled temperature (23 ± 2°C) and under a 12 h light/dark cycle. Animals were acclimatized to the new conditions for 7 days.

The experimental protocol was approved by the National Institute of Ethics Committee at Sichuan Agricultural University (approval number SYXK (Sichuan) 2014-187). The humane endpoints were a weight loss above 15% of initial weight or animals in a state of prostration. Animals that reach one of these endpoints were euthanized by cervical dislocation by caretakers [28]. All efforts were made to minimize suffering of animals.

### 2.5. The High Temperature Stability Test of GOS

This method conformed to the Compilation of Technical Guidance for Veterinary Drug Research [29]. Briefly, three units of GOS (containing 15% of GTE) were stored in climate cabinets at 60 ± 2°C for 10 days. Samples were stored in brown glass containers and the tannin content was analyzed at 5 and 10 days.

### 2.6. The Accelerated Stability Test of GOS

The accelerated test is used to calculate the stability of liquid traditional drugs in the Compilation of Technical Guidance for Veterinary Drug Research [29]. Four units of GOS (containing 15% of GTE) were stored in climate cabinets at 40 ± 2°C, and then at determined times (0, 30, 60, 90, and 180 days after conditioning), solutions were investigated with visual inspection, pH measurement, and microbiological examination, according to the methods mentioned in the Chinese Pharmacopoeia [25].

### 2.7. Acute Toxicity of* Galla Chinensis* Oral Solution

The oral acute toxicity of GOS was calculated by the 50% lethal dose (LD_50_). Throughout this test, animals were dosed only once at a time. In brief, adult male and female mice were divided into five groups of 10 mice (5 females and 5 males); each group was treated by gavage with GOS at doses of 2700, 3600, 4500, 5400, and 6300 mg/kg body weight. The volume of GOS was 10 mL/kg in each case. The food was provided for a further 3-4 h, and the animals were observed for behavioral changes, signs of toxicity, and death during the first 12 hours and thereafter twice a day for 14 days. The LD_50_ was calculated according to the improved method described by Xiang et al. [30]. In this study, animals obviously in pain, showing signs of severe and enduring distress, or characterized as moribund should be humanely killed rather than being allowed to survive to the end of the scheduled study [31].

### 2.8. Antidiarrheal Activity

Male ICR mice (weighing 18–22 g) were divided into six groups. Each group contained five animals. All of them were fasted for 8 h before pretreatment. Groups one (blank control) and two (negative control) got normal saline. Groups three, four, and five received different doses of GOS (5%, 10%, and 15% of GTE, resp.) at a dose of 10 mL/kg. Group six served as positive control and received loperamide (10 mg/kg) which was suspended in distilled water.

After 30 min, animals were treated with 0.2 mL of castor oil orally, except those in group one which received equal normal saline instead. Mice were placed in separate boxes with clean paper for antidiarrheal activity evaluation. The paper in each box was replaced every hour.

Mice were observed for 4 h. The frequency of droppings and the score of stools were noted. The grading system followed the method previously used [32, 33]. Briefly, score 0 means no diarrhea (solid stool); score 1 means soft but formed stool; score 2 means very soft stool; score 3 means diarrhea (liquid stool). The score of each animal was calculated by the following formula:

(2) Individual  score=1×no.  score  1+2×no.  score  2+3×no.  score  3.

Then, the scores of individuals in each group were added together and the average score was used to calculate a reduction of diarrhea by the following formula [9]:

(3) $$\begin{array}{c} Diarrhea\;\;reduction=\frac{Mean\;\;castor\;\;oil\;\;diarrheal\;\;score\;\;\left(negative\;\;control\right)-mean\;\;score\;\;of\;\;treatment\;\;group}{mean\;\;castor\;\;oil\;\;diarrheal\;\;score\;\;\left(negative\;\;control\right)}\times 100\%. \end{array}$$

### 2.9. Antisecretory Effect

The fluid in the small intestine is also an index to evaluate the antidiarrheal activity. The antisecretory effect was evaluated using the method described by Mehmood et al. [34] with slight modification. Briefly, male ICR mice (weighing 18–22 g) were divided into six groups of five animals each. Mice were fasted for 12 h. Groups one and two received normal saline, whereas groups three, four, and five got various concentrations of GOS (10 mL/kg, resp.). Group six received loperamide (10 mg/kg). Mice in each group got 0.15 mL of liquid orally. 30 min later, mice were treated with 0.2 mL of castor oil by gavage, except those in group one. After 30 min, mice of all groups were sacrificed by cervical dislocation. The whole small intestines were moved out and the fluid accumulation ratio was calculated according to the following formula [34]:

(4) Fluid  accumulation  ratio=Small  intestine  weightgmouse's  Body  weightg×100%.

### 2.10. Intestinal Motility Study

Thirty mice were fasted for 12 h and divided into 6 groups as described above. Normal and negative controls got normal saline. Test groups received three different concentrations of GOS (10 mL/kg, resp.). Positive control got loperamide (10 mg/kg). After 30 min, except for the normal control, each animal had 0.2 mL of castor oil by gavage. A further 30 min later, mice received 0.3 mL of charcoal meal (5% deactivated charcoal in 10% aqueous tragacanth) [35]. Then, all mice were sacrificed by cervical dislocation after 30 min. The bodies were opened and the small intestines were moved out. The total length of the small intestines and the moving distance of charcoal meal in the guts were measured. Intestinal motilities were evaluated by the percentage [9, 35] which is called moving distance inhibition rate:

(5) Distance  inhibition=length  of  charcoal  meallength  of  small  intestine×100%.

### 2.11. Data Analysis

The results in this study are expressed as means ± SD. And data were analyzed by one-way analysis of variance (ANOVA). The differences between groups would be considered significant when values of *P* < 0.05.

## 3. Results

### 3.1. Determination of Tannin Content in GTE

The concentrations of gallic acid equivalents to obtain the calibration curve ranged from 0.02 to 0.1 mg/mL. The absorbance of phenols in GTE was within the concentration range of the curve, so it could be used for the determination of the tannin content. There was a linear relationship between concentrations of gallic acid and absorbance at 760 nm; the linear equation was *y* = 86.729*x* + 0.0529 with a coefficient of determination of *R*^2^ = 0.9991. Total phenolic content and nontannin polyphenol content were determined as gallic acid equivalents. Tannin content was the difference between total phenolic content and nontannin polyphenol content. The results are listed in Table 1. The contents of total phenolics and tannins in each gram of GTE were 66.16% and 47.75%, respectively.

### 3.2. The High Temperature Stability Test of GOS

According to the Compilation of Technical Guidance for Veterinary Drug Research, if the drug content here does not decrease by more than 5% every 5 days, it can be considered as a nonsignificant decrease [29]. The contents of total phenolics and tannins every 5 days are shown in Table 2. The tannin content of GOS reduced by 3.72% and 1.02% on the first and second 5 days, which was lower than the blank control (BC, prepared only with the tannin extract, purified water) that measured 4.98% and 11.37%. The decrease percentage of GOS tannin content was 4.71% in the whole 10 days, which could be considered as a nonsignificant decrease. These results suggested that GOS had a greater stability than BC under this condition.

### 3.3. The Accelerated Stability Test of GOS

Throughout this study, no changes in color and clarity were observed in any of the samples. There was no appearance of any visible particulate matter or haziness. The pH of samples did not vary by more than 0.2 pH units from day 0 pH (3.3 ± 0.1). No sample showed any signs of microbial growth at day 0, day 30, day 60, day 90, or day 180. The contents of samples at the determined times are shown in Table 3. According to the results, solutions did not change by more than 8.2% at 40 ± 2°C after six months, and the 95% confidence interval was 90.39%, 101.41%.

### 3.4. The Acute Toxicity of GOS

The animals showed no behavioral changes, signs of toxicity, or death even in the highest-dose group (6300 mg/kg) after 14 days of observation. According to the guiding principles for veterinary medicine research technology assembler, the drug is considered as practically nontoxic and its LD_50_ is estimated to be higher than 5000 mg/kg. So, the LD_50_ of GOS was determined to be more than 5000 mg/kg.

### 3.5. Antidiarrheal Activity

**Figure 1:**
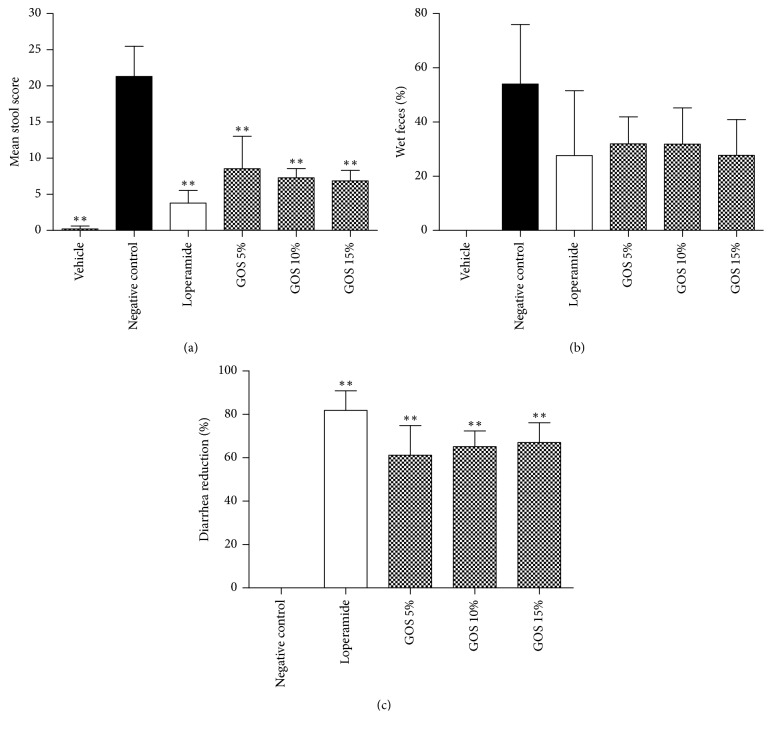
Antidiarrheal activity of different doses of GOS (10 mL/kg) as well as loperamide (10 mg/kg) in castor oil-induced diarrhea of mice. (a) Average score of stools in each group, (b) percentage of wet feces in the groups, and (c) reduction rate of diarrhea compared with the negative control. ^*∗∗*^*P* < 0.01, compared with the negative control.

In the present research, there was clinical diarrhea apparent for the next 4 h in the castor oil-treated groups. Pretreatment with various doses of GOS could lessen the diarrhea symptoms dose-dependently. The effects of GOS on the mean stool score (Figure 1(a)), percentage of wet feces (Figure 1(b)), and the diarrhea reduction (Figure 1(c)) are shown in Figure 1.

As a result, all kinds of doses of GOS reduced the score of stools in test animals compared with the negative control. There was no significant difference between the positive control (loperamide) and the GOS groups, indicating that the GOS had equal effect on treating diarrhea as loperamide in mice (Figure 1(a)). Meanwhile, decreases were observed in the percentage of wet feces, reflecting that the GOS could reduce the amount of wet feces (Figure 1(b)). Diarrhea reduction indicated that various doses of GOS were able to inhibit castor oil-induced diarrhea in mice and showed dose-dependent resistance. Compared with loperamide, orally administered GOS of 10% and 15% GTE showed no significant difference in diarrhea reduction (Figure 1(c)).

### 3.6. Antisecretory Effect on the Small Intestine

**Figure 2:**
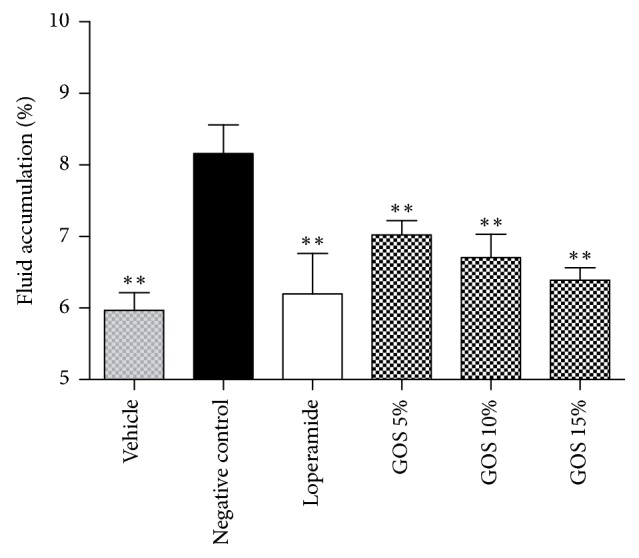
Antisecretory effect on the small intestine at different doses of GOS (10 mL/kg) as well as loperamide (10 mg/kg) in castor oil-induced diarrhea in mice. ^*∗∗*^*P* < 0.01, compared with the negative control.

As shown in Figure 2, GOS and loperamide could significantly inhibit the fluid in the small intestines in mice when compared with the negative control. GOS in the test groups reduced the fluid secretion dose-dependently and had no significant difference with the positive control, demonstrating that GOS could decrease the fluid secretion of castor oil-induced diarrhea in mice.

### 3.7. Intestinal Motility Study

**Figure 3:**
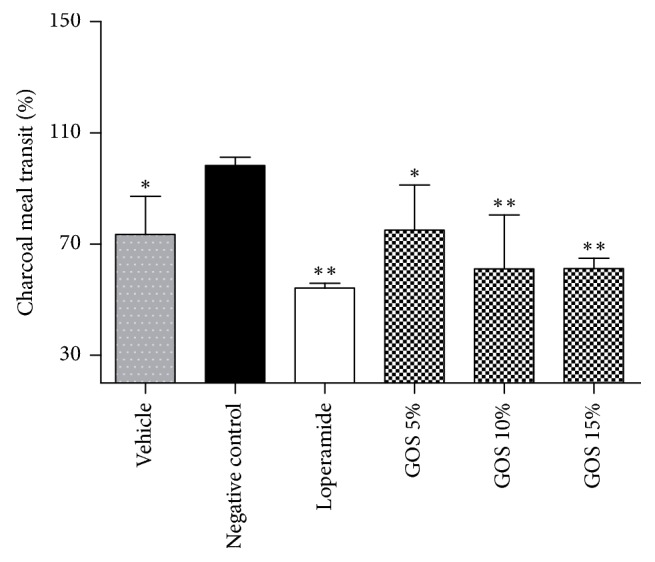
Effects of different doses of GOS (10 mL/kg) and loperamide (10 mg/kg) on intestinal motility in castor oil-induced diarrhea in mice. ^*∗∗*^*P* < 0.01 and ^*∗*^*P* < 0.05, compared with the negative control.

There was an increase in the intestinal motility after treatment with castor oil. Here, we used the charcoal meal transit to find out whether the GOS causes a decrease in intestinal motility. As expected, the oral administration of GOS and positive drug loperamide exhibited significant decreases of charcoal meal transit obviously compared to the negative group, which means GOS and loperamide pretreatment inhibited the castor oil-induced increase in the intestinal motility. There was no significant difference between GOS and loperamide. A dose-dependent manner of GOS was also observed in this study (Figure 3).

## 4. Discussion

It is well known that traditional herbal medicines can treat or mitigate diarrhea.* Galla Chinensis* is one of the herbs that is still used in China to treat diarrhea, and its antidiarrheal activity also has been described in the Chinese Pharmacopeia [25]. However, hydrolyzable tannin, the main component of* Galla Chinensis*, can easily be hydrolyzed and oxidized [27], Therefore, in this study, we made a pharmaceutical preparation of* Galla Chinensis* tannin extract to solve this problem and studied its acute toxicity and antidiarrheal activity in mice.

We found that GOS could endure high temperature without a significant decrease of tannin content. In the accelerated stability test, after being stored at 40 ± 2°C for six months, the tannin content of GOS was still more than 90%. The LD_50_ test suggested that GOS was practically nontoxic. Meanwhile, GOS showed significant antidiarrheal activity in a castor oil-induced diarrhea model in mice.

In the accelerated stability study, the tannin content of GOS was still more than 90%, and the pH did not change by more than 0.2 pH units after being stored at 40 ± 2°C for six months. pH level is a strong determinant in the fate of tannins [36], which might be the main reason for the fewer reductions of tannin content.

GOS could reduce the diarrhea symptoms (mainly wet feces) dose-dependently in mice. The antidiarrheal activity of GOS was similar to that of the positive drug. Excessive secretion and motility in the small intestines are the major characteristics of secretory diarrhea [5]. So, the antisecretory and antimotility activities play vital roles in evaluating antidiarrheal mechanisms of drugs. The current studies showed that GOS exhibited resistance to fluid secretion and intestinal motility. The results reflected that GOS at high dose (15% GTE) had the highest antisecretory and antimotility activities. The antidiarrheal activities about the traditional use of* Galla Chinensis* had also been explained.

It is believed that the cystic fibrosis transmembrane conductance regulator (CFTR) as the major Cl^−^ channel is responsible for fluid secretion in diarrheas caused by bacterial enterotoxins [37]. Therefore, CFTR inhibitors have the potentials of antidiarrheal therapy. It is confirmed that a hydrolyzable tannin extracted from* Galla Chinensis* is a new class of CFTR inhibitors [13]. So, it is supposed that GOS might resist the fluid secretion by acting as CFTR inhibitors.

On the other hand, previous studies have reported that antimotility and antidiarrheal activities of medicinal plants are due to tannins [38]. It is known that the tannin-rich plant* Terminalia bellirica* offers a combination of anticholinergic and calcium antagonist properties, which explain its folkloric use in colic, diarrhea, and asthma [39]. Meanwhile, various medicinal plants have been found to have spasmolytic activities, which were due to the blockade of the Ca++ channels [40], such as* Terminalia bellirica *[39],* Salvia ballotiflora *[41], and* Cissampelos sympodialis *Eichl. [4], and they all have demonstrated antidiarrheal activities. Hence, we considered that GOS might affect gastrointestinal motility like loperamide and atropine through anticholinergic activity, or spasmolytic activity by blocking Ca++ channels in the small intestinal smooth muscle.

In conclusion, GOS was a stable preparation for tannins and exhibited antidiarrheal properties. The drug was determined to be nontoxic, and it exhibited antidiarrheal activity against castor oil-induced diarrhea in mice through alleviating diarrhea symptoms and inhibiting small intestinal motility and secretion. These results suggested that GOS is an effective preparation, which is expected to be applied in clinical settings as an antidiarrheal drug.

## Figures and Tables

**Table 1:** The contents of total phenolics, nontannin polyphenols, and tannins in GTE.

	OD_760_	Content (S)	Content (G)	Percent
Total phenolics	2.81 ± 0.04	0.0318 ± 0.0004	661.65 ± 8.61	66.16%
Nontannin polyphenols	0.82 ± 0.01	0.0088 ± 0.0001	184.11 ± 1.55	18.41%
Tannin		0.0229 ± 0.0004	477.54 ± 9.17	47.75%

The unit of contents was milligram (mg). Results are expressed as means ± standard deviation (*n* = 3). S represents the contents of phenols in the sample, and G represents the contents of phenols in each gram of GTE. Percentage refers to the proportion of the various ingredients in each gram of GTE.

**Table 2:** The results of the high temperature stability test of GOS.

	Day 0	Day 5		Day 10	
	BC/GOS	BC	GOS	BC	GOS
Total phenolic content	99.25 ± 1.29	91.87 ± 0.24	93.67 ± 1.34	94.58 ± 0.11	94.74 ± 0.09
Tannin content	71.63 ± 1.38	68.06 ± 1.21	68.96 ± 1.25	60.32 ± 0.06	68.26 ± 0.50
Concentration (% of day 0 tannin measurement)	100	95.02 ± 1.69	96.28 ± 1.75	84.21 ± 0.09	95.29 ± 0.70

The unit of contents was milligram per milliliter (mg/mL). Results are expressed as means ± standard deviation (*n* = 3).

**Table 3:** The results of the accelerated stability test of GOS.

	Day 0	Day 30	Day 60	Day 90	Day 180
Total phenolic content	99.25 ± 1.29	95.48 ± 1.34	93.84 ± 0.10	93.78 ± 0.96	93.22 ± 0.13
Tannin content	71.63 ± 1.38	70.77 ± 1.25	68.42 ± 0.07	69.83 ± 2.22	65.76 ± 0.18
Concentration (% of day 0 tannin measurement)	100	98.79 ± 1.75	95.52 ± 0.10	97.49 ± 3.10	91.80 ± 0.25

The unit of contents was milligram per milliliter (mg/mL). Results are expressed as means ± standard deviation (*n* = 4).
